# Empirical Mode Decomposition and Neural Networks on FPGA for Fault Diagnosis in Induction Motors

**DOI:** 10.1155/2014/908140

**Published:** 2014-02-11

**Authors:** David Camarena-Martinez, Martin Valtierra-Rodriguez, Arturo Garcia-Perez, Roque Alfredo Osornio-Rios, Rene de Jesus Romero-Troncoso

**Affiliations:** ^1^HSPdigital-CA Mecatronica, Facultad de Ingenieria, Universidad Autonoma de Queretaro, Campus San Juan del Rio, Rio Moctezuma 249, 76807 San Juan del Río, QRO, Mexico; ^2^HSPdigital-CA Telematica, Procesamiento Digital de Señales, DICIS, Universidad de Guanajuato, Carr. Salamanca-Valle km 3.5 + 1.8, Palo Blanco, 36700 Salamanca, GTO, Mexico

## Abstract

Nowadays, many industrial applications require online systems that combine several processing techniques in order to offer solutions to complex problems as the case of detection and classification of multiple faults in induction motors. In this work, a novel digital structure to implement the empirical mode decomposition (EMD) for processing nonstationary and nonlinear signals using the full spline-cubic function is presented; besides, it is combined with an adaptive linear network (ADALINE)-based frequency estimator and a feed forward neural network (FFNN)-based classifier to provide an intelligent methodology for the automatic diagnosis during the startup transient of motor faults such as: one and two broken rotor bars, bearing defects, and unbalance. Moreover, the overall methodology implementation into a field-programmable gate array (FPGA) allows an online and real-time operation, thanks to its parallelism and high-performance capabilities as a system-on-a-chip (SoC) solution. The detection and classification results show the effectiveness of the proposed fused techniques; besides, the high precision and minimum resource usage of the developed digital structures make them a suitable and low-cost solution for this and many other industrial applications.

## 1. Introduction

Many industrial processes involve the use of induction motors that are required to operate in optimal conditions; then, the diagnosis of induction motors becomes a relevant task because the presence of faults can lead to setbacks and substantial economical losses [1]. The diagnosis of induction motor faults can be treated as a problem of pattern recognition, mainly when there is the possibility of different faults, as it may happen in real industrial applications. The solution to this problem can be divided into three stages, beginning with the processing of the monitored signal; then, performing the extraction of relevant features that indicate the presence of a motor fault from the processed signal; and the identification and classification of the motor condition based on the extracted features. The signal processing task is very challenging since the monitored signals, such as currents, voltages, and vibrations among others, present non-stationary features disturbing the results of the classical processing techniques [2, 3]. Moreover, the feature extraction and selection have to be carried out carefully since a large number of feature parameters may increase the computational load and deteriorate the identification capability of a subsequent classifier. Therefore, it is necessary to have a system that can process non-stationary signals, extract the relevant features from these signals, and provide the condition classification without compromising the online operation in order to automatically diagnose faults in induction motors. Regarding the implementation of the aforementioned system, a promising technology for online operation is the field programmable gate arrays (FPGA), thanks to the natural parallelism and high-performance, especially for implementing neural computing algorithms as it has been demonstrated in a number of industrial applications [4, 5].

Several techniques have been used for non-stationary signal processing. The short-time Fourier transform (STFT) is used in [6] to obtain a time-frequency spectrum decomposition of the signal, assuming that it is linear and stationary in the analyzed time window, which is not true in transient phenomena. Another technique is the wavelet transform (WT) [7]; however, the mother wavelet has to be selected appropriately and prudently since the contents of its daughter wavelets have to be largely similar to the analyzed signal in order to ensure suitable results. The Wigner-Ville distribution (WVD) has also been used; nevertheless, the weakness of this method is the presence of cross terms, indicated as negative amplitudes for some frequency ranges. Besides, WVD suffers of the aliasing problem [8]. A high-resolution spectral analysis through multiple signal classification (MUSIC) is presented in [9]; yet, the frequencies of interest in the analyzed signal have to be known or at least supposed *a priori*, which is not possible for a number of applications. The Hilbert-Huang transform (HHT) is also a time-frequency analysis technique composed of two main parts: the empirical mode decomposition (EMD), and the Hilbert transform (HT) [10]. The EMD is an intuitive, unsupervised, and self-adaptive method that can decompose a non-stationary and nonlinear signal into narrowband oscillatory components called intrinsic mode functions (IMF). These advantages have allowed its effective use in many industrial applications such as noise reduction [11], fault diagnosis in bearings [12–16], broken rotor bars [17–21], and rotor eccentricities [22–25]. Despite the potential applications, most of the related works using EMD require a personal computer (PC) applied to offline signal processing, and this is due to the complexity of the technique as well as to its high computational load. Nevertheless, the spread use of the EMD technique makes it a potential necessity for hardware implementation in order to create online processing systems. Some online processing systems have been presented in [26, 27]; yet, they are based on the FFT which limits their application to stationary signals.

Few works have implemented partially or totally the EMD method in hardware. In [28], a combination of software-hardware for EMD implementation is proposed, but this solution prompts high costs in logic elements and low processing speed. Another combination of FPGA and digital signal processor (DSP) is presented in [29], where the whole EMD process is done by the DSP, and the FPGA is only used to control the data flow among the analog to digital converter (ADC), digital to analog converter (DAC), and the system memory. The inconvenience of this implementation is also the speed limitation because its operating frequency is below 1 kHz. A real-time FPGA implementation is presented in [30]; however, this application approximates the spline-cubic interpolation with a linear sawtooth function in order to simplify the computational load of calculating signal envelopes. This is a problem since it does not produce a smooth signal, and as an aftereffect, a leaking of high-frequency components rides into each residue function [31], modifying the results of subsequent IMFs. In this context, a complete FPGA implementation of the EMD that does not present the problem of high-frequency components rides is missing.

Despite the efforts, there is a need for the development of digital structures that combine several processing methodologies to present online solutions for complex problems such as induction motor fault detection, namely, to combine time-frequency decomposition analysis of non-stationary signals, a feature extraction module, and an automatic classifier.

This paper presents a novel digital structure to implement the EMD method in FPGA using the full spline-cubic function and not just a linear approximation, taking into account that the spline-cubic function does not present the problem of high-frequency component rides, making it more suitable for time-frequency processing. This novel structure for the hardware computation of EMD method is combined with an ADALINE-based frequency estimator and an ANN classifier in the same FPGA to provide an intelligent methodology for online multiple fault diagnosis in induction motors. First, the startup transient current signal, which is non-stationary, is processed by the EMD in order to obtain the IMFs; then, the ADALINE carries out the amplitude estimation of these IMF frequency components. Finally, these frequency components are used as input features of an ANN classifier for the automatic diagnosis of motor faults such as one (1BB) and two broken rotor bars (2BB), bearing defects (BD), and unbalance (UNB). Besides, the overall methodology implementation into an FPGA allows an online and real-time operation, thanks to its parallelism and high-performance capabilities as a system-on-a-chip (SoC) solution. Furthermore, the developed EMD digital structure can also be used to solve other problems requiring online and real-time processing capabilities.

## 2. Theoretical Background

### 2.1. Fault Detection in Induction Motors

The identification and classification of multiple faults in induction motors are very important since in real life rotating machines can be affected by several faults, where 50% of these faults are bearing related, 10% are rotor faults, and unbalance is within the 12% of other faults [2]. The automatic identification and classification of the induction motor condition may be provided by artificial intelligent techniques such as artificial neural networks (ANN), which have been established as a powerful tool in the condition identification of rotating machinery [32]. The condition identification through ANNs of different faults has also been presented [6, 33–35]. In [6], shorted turns and power supply imbalance faults are analyzed through vibration signals. Faults related to stator winding, inter-turn short, and rotor dynamic eccentricity are classified by the current signal information [33]. Current and vibrations signals are analyzed in order to classify different bearing faults [34]. Other faults such as broken rotor bars and broken end rings are also classified by extracting current signal features [35]. Although ANNs have been successfully applied for condition identification of motor faults, the online implementation remains as a challenge since it requires a processing technique that gives relevant information in order to extract features related to the different faults; moreover, if the number of features is large, the classifier complexity may increase. As a result, a compromise must be established between the number of features that feed the classifier and the overall computational load required for online operation.

**Figure 1:**
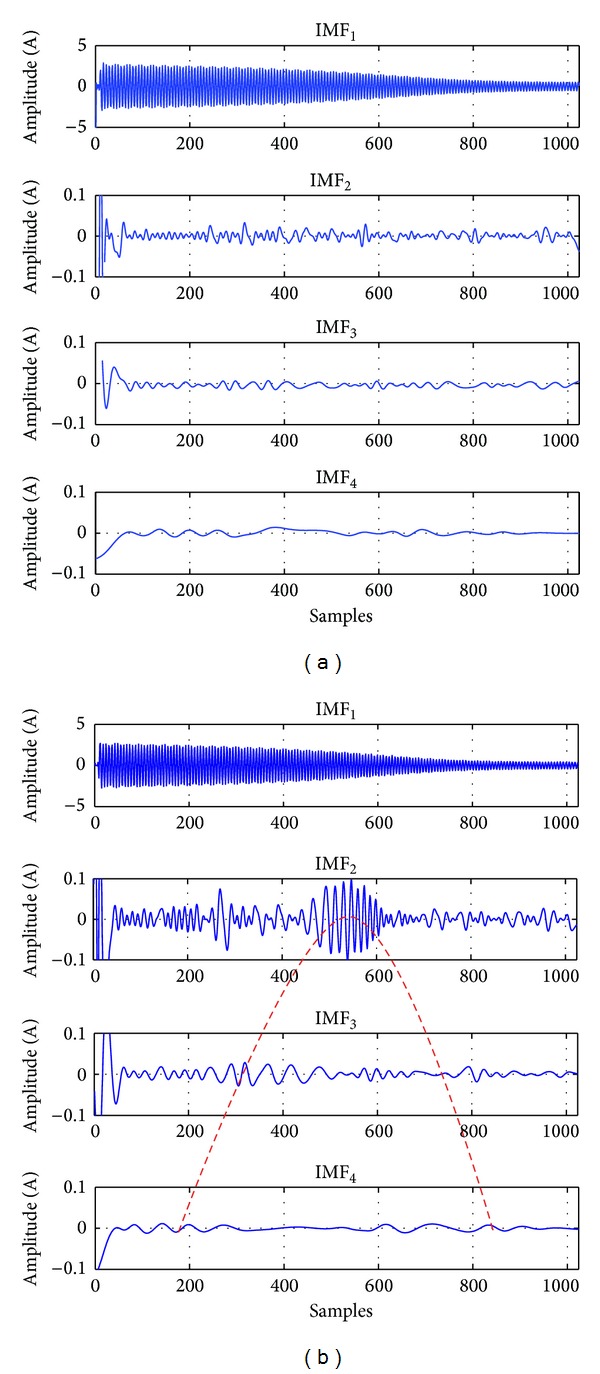
IMF signal decomposition for a current signal of an induction motor: (a) healthy motor and (b) faulty motor with two broken rotor bars.

In general, motor currents and voltages are non-stationary signals and their temporal properties are influenced by many factors, including electrical power supply, load variations, noise, motor geometry, and fault conditions. When an induction motor is started up from standstill, electromagnetic transients (EMT) take place regardless of the motor condition. The EMT provoke low-frequency oscillations in the IMF signal decomposition, and those are usually contained in the upper IMFs, as can be observed in Figure 1, where the evolution in time of low-frequency components is notoriously higher for a faulty motor.

Motor current signals contain spectral components which vary over time, and the fault signatures are revealed through the distortion of these components. In [36], the wavelet packet analysis was used to process the motor current signals, where the wavelet packet transform decomposes the signal utilizing both its low- and high-frequency components. The broken rotor bar fault signature was extracted in the low frequencies by using the instantaneous amplitude of stator current [37], and the DWT is used as an efficient time-scale algorithm, which gives optimal frequency accuracy at low-frequency bandwidth (1.2 Hz–9.6 Hz) [38].

The fault indicator proposed in this paper to detect mechanical and electric faults in induction motors is based on the observation of the startup motor current that is distorted in the presence of these faults. Consequently, in the presence of such faults, the spectral components in the current increase when compared to a healthy spectrum. Therefore, current spectrum variations provide some clues to notice the presence of mechanical and electrical faults. Relative changes in the low frequencies, as would be seen through the processing of the startup current, appear promising for detecting changes in the induction motor condition when the induction motor startup current is non-stationary.

### 2.2. Empirical Mode Decomposition

EMD is an adaptive and efficient method introduced by Huang et al. [10] to decompose nonlinear and non-stationary signals into intrinsic mode functions (IMF). The process for obtaining the IMF decomposition is known as “sifting,” with the following steps.

**Step 1**

Identify all the local maxima and minima of the signal.

**Step 2**

Connect all the local maxima by using spline-cubic interpolation to create the upper envelope. Repeat the procedure on the local minima to create the lower envelope.

**Step 3**

Designate the mean of the upper and lower envelopes as *m*_1_.

**Step 4**

Calculate the difference *h*_1_ between the original signal *x*(*t*) and *m*_1_ as the first component:

(1) $$\begin{array}{c} h_{1}=x\left(t\right)-m_{1}. \end{array}$$

**Step 5**

Verify if *h*_1_ satisfies the conditions of the IMF or a criterion to define an IMF; take it as the first IMF of *x*(*t*). But if *h*_1_is not an IMF, treat it as a proto-IMF and name it as *h*_11_. Take *h*_11_ as the original signal and repeat the first four steps until *h*_1*k*_ satisfies the conditions of IMF, and designate it as *c*_1_.

(2) $$\begin{array}{c} c_{1}=h_{1k}, \end{array}$$

where *k* indicates the number of iterations to produce an IMF. The standard deviation (SD) criterion can be used to determine when the signal *h*_1_ is an IMF, which is defined as

(3) $$\begin{array}{c} {\text{SD}}_{k}=\frac{\sum_{t=0}^{T}{\left|h_{k-1}\left(t\right)-h_{k}\left(t\right)\right|}^{2}}{\sum_{t=0}^{T}h_{k-1}^{2}}. \end{array}$$

**Step 6**

Subtract *c*_1_ from the original signal *x*(*t*) by

(4) $$\begin{array}{c} x\left(t\right)-c_{1}=r_{1}. \end{array}$$

**Step 7**

Treat *r*_1_ as the original signal and apply Steps 1 to 6 for obtaining the other IMFs, *c*_2_, *c*_3_,…, *c*_*n*_ as follows:

(5) $$\begin{array}{c} r_{1}-c_{2}=r_{2}\\ \vdots \\ r_{n-1}-c_{n}=r_{n}. \end{array}$$

The decomposition process can be stopped when *r*_*n*_ becomes a monotonic function from which no more IMF can be extracted. However, it is well known that only a determined number of IMFs have physical meaning and it is only necessary to take a certain number of IMFs to extract the relevant information from the original signal. At the end of the process it gives

(6) $$\begin{array}{c} x\left(t\right)=\overset{n}{\underset{i=1}{\sum }}c_{i}\left(t\right)+r_{n}, \end{array}$$

where the signal *x*(*t*) is decomposed into *n* intrinsic modes and a residue *r*_*n*_.

### 2.3. Adaptive Linear Network

The fault feature extraction is a procedure for obtaining parameters that represent the induction motor condition in order to achieve a future fault classification. Different parameters have been reported for this application; for instance, statistical parameters such as the standard deviation, the local maxima and minima values, and the skewness and the kurtosis coefficients have been extracted from the motor input current [33]. In addition to some statistical parameters, the outer race, inner race, and ball spinning fault frequency components are also extracted from vibration and current signals [34]. The frequency spectra of vibration and current signals have also been computed [6, 35], respectively, where the amplitudes of some frequency components are extracted. Nevertheless, in non-stationary signals some of the aforementioned parameters, mainly the frequency components, may change through time producing a wrong classification result. A suitable solution for frequency component estimation of non-stationary signals is the use of an adaptive linear neural network (ADALINE) as has been demonstrated in [39]; besides, its inherent parallel performance makes it also attractive for FPGA implementation.

ADALINE is an adaptive filter used for extracting signals from noisy environments and for model identification as well as for tracking and estimating frequency components [39]. For the last application it is assumed, in concordance with the Fourier series, that a signal is the sum of all frequency components with unknown amplitudes and phase angles. Therefore, the representation of a signal *y* is

(7) y(k)=∑m=1MAmsin(2πfmkΔt+ϕm)=∑m=1M(Amcos⁡ϕmsin2πfmkΔt   +Amsinϕmcos⁡2πfmkΔt)=∑m=1M(amsinθm+bmcos⁡θm),

where *A*_*m*_ and *φ*_*m*_ are the amplitudes and phase angles of *m*th frequency component, respectively; *M* is the total frequency components, *θ*_*m*_ = 2*πf*_*m*_ *k*Δ*t*, *k* is the sampling index, Δ*t* is the sampling interval, *a*_*m*_ = *A*_*m*_cos⁡*φ*_*m*_, and *b*_*m*_ = *A*_*m*_sin*φ*_*m*_. Equation (7) can be rewritten as

(8) $$\begin{array}{c} y\left(k\right)=w^{T}\left(k\right)\cdot x\left(k\right) \end{array}$$

with

(9) $$\begin{array}{c} w\left(k\right)={\left[\begin{array}{ccccc} a_{1} & b_{1} & \cdots & a_{m} & b_{m} \end{array}\right]}^{T},\\ x\left(k\right)={\left[\begin{array}{ccccc} \sin\theta_{1} & \cos\theta_{1} & \cdots & \sin\theta_{m} & \cos\theta_{m} \end{array}\right]}^{T}. \end{array}$$

**Figure 2:**
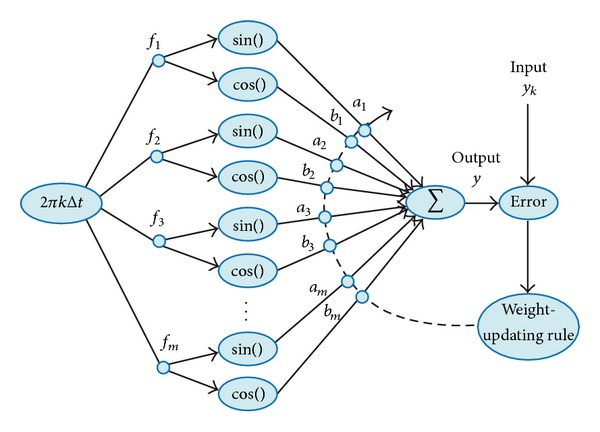
ADALINE block structure for frequency estimation.

For applications on frequency estimation, ADALINE is arranged as shown in Figure 2. The initial guess of **w** is a zero vector; then, its elements are adjusted during each sample through a weight-updating rule to minimize the error or difference between the estimated output *y* and the real one *y*_*k*_. Simultaneously, the amplitude *A*_*m*_ and phase angle *φ*_*m*_ of the *m*th frequency component are computed as follows:

(10) $$\begin{array}{c} A_{m}=\sqrt{a_{m}^{2}+b_{m}^{2}}\;\;\varphi_{m}=\text{ta}{\text{n}}^{-1}\left(\frac{a_{m}}{b_{m}}\right), \end{array}$$

where −*π* ≤ *φ*_*m*_ ≤ *π*.

The error is equal to zero when all the frequency components of *y*_*k*_ are modeled by ADALINE.

The weight-updating rule used to minimize the error is the least mean squares (LMS), which reduces the mean squared error (MSE) defined by

(11) $$\begin{array}{c} E_{k}^{2}=\frac{1}{2L}\overset{L}{\underset{k=1}{\sum }}\varepsilon_{k}^{2}, \end{array}$$

where *L* is the number of analyzed samples and *e*_*k*_ is the difference between the ADALINE output *y* and the desired output *y*_*k*_ given in (12):

(12) $$\begin{array}{c} \varepsilon_{k}=y_{k}-y. \end{array}$$

Finally, the modification on the weights is given in (13), where *α* is a constant of proportionality, known as the ADALINE learning rate:

(13) $$\begin{array}{c} w\left(k+1\right)=w\left(k\right)+\alpha \varepsilon_{k}x\left(k\right). \end{array}$$

### 2.4. Artificial Neural Networks

**Figure 3:**
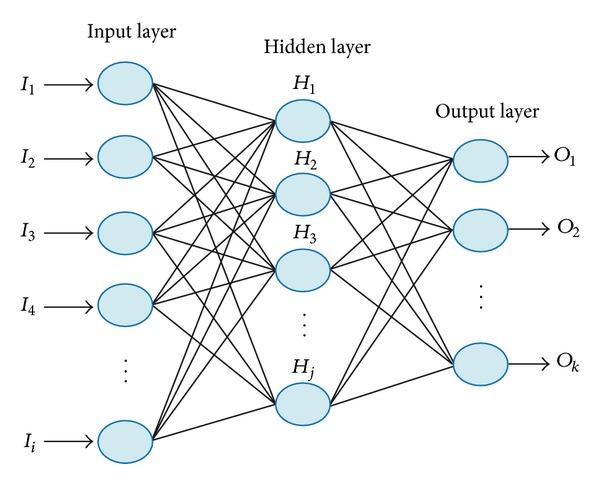
Architecture of a FFNN.

ANNs are computational models that simulate the neurological structure of the human brain and its capability to learn and solve problems through pattern recognition for industrial applications ranging from metal removal prediction [40] up to induction motor diagnosis [41]. There are different ANN architectures such as feed-forward networks (FFNN), recurrent networks, feedback networks, radial basis function networks, and Kohonen self-organizing map networks, among others. The most popular architecture for ANN is the FFNN since it is simple and practical as a classifier and because it has a low computational load. FFNN is characterized by having a layered architecture with single or multiple neurons in each layer [41], as shown in Figure 3. The mathematical model describing each neuron is given in (14), where *y*, *ω*_*i*_, *x*_*i*_, *b*, *f*(·), and *I* are the output, weights, inputs, bias, activation function, and the total number of inputs, respectively. The FFNN model consists in the sum of products between the inputs and their associated multipliers, commonly called weights, plus a bias. Then, this result is evaluated by a nonlinear function to provide the NN with the ability to model nonlinear relationships. In this architecture, the information flows in one direction only, from the input layer, through the hidden layer, up to the output nodes. To characterize the network weights, pairs of input-output data are presented; then, a training rule for adjusting these weights is used. The training process minimizes the error between the desired and the calculated outputs and it is repeated until the overall error is acceptable:

(14) $$\begin{array}{c} y=f\left(\overset{I}{\underset{i=1}{\sum }}\omega_{i}x_{i}+b\right). \end{array}$$

## 3. Proposed Methodology and Its FPGA Implementation

This section presents the overall methodology and its FPGA implementation in order to provide an online diagnosis of an induction motor with different conditions such as one broken rotor bar (1BB), two broken rotor bars (2BB), bearing defect (BD), unbalance (UNB), and healthy (HLT), where the proposed methodology contains three processing stages. First, the EMD algorithm is used to separate the different low-frequency components to estimate the fault indicators; second, ADALINE estimates their magnitudes; and third, an ANN is used to classify the motor faults.

### 3.1. Overall Methodology

**Figure 4:**
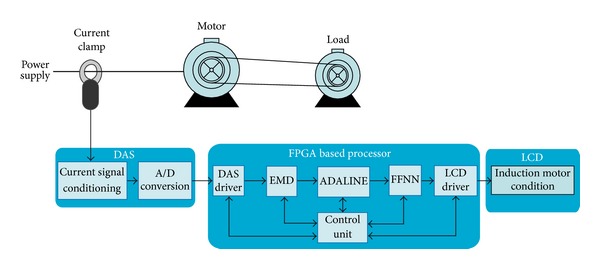
Proposed methodology for fault diagnosis in induction motors.

The overall methodology is shown in Figure 4. First, the system uses a current clamp to measure one phase of the stator current and a data-acquisition system (DAS) to condition and quantize the signal. Then, the discrete signal is passed through the FPGA-based processor for automatic diagnosis, where an overall control unit coordinates the following actions: the DAS driver for data acquisition, the EMD processing unit to compute the IMFs, the ADALINE unit for feature extraction, and finally, the FFNN unit that accomplishes the classification. Then, the result of the induction motor condition is displayed to the user.

### 3.2. FPGA-Based Processor

**Figure 5:**
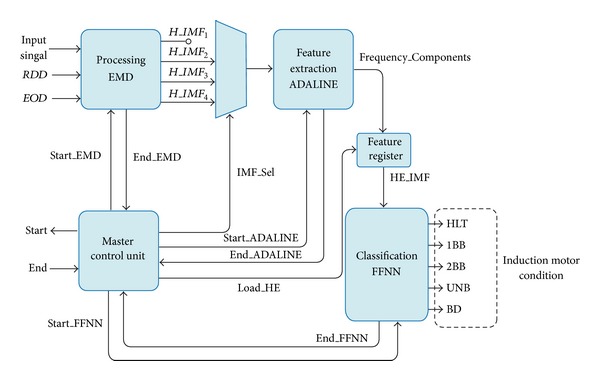
FPGA-based processor.

**Figure 6:**
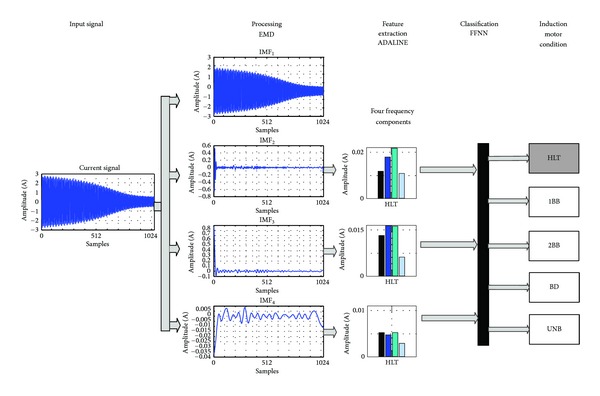
Proposed methodology flow up, showing a healthy induction motor condition diagnosis.

**Figure 7:**
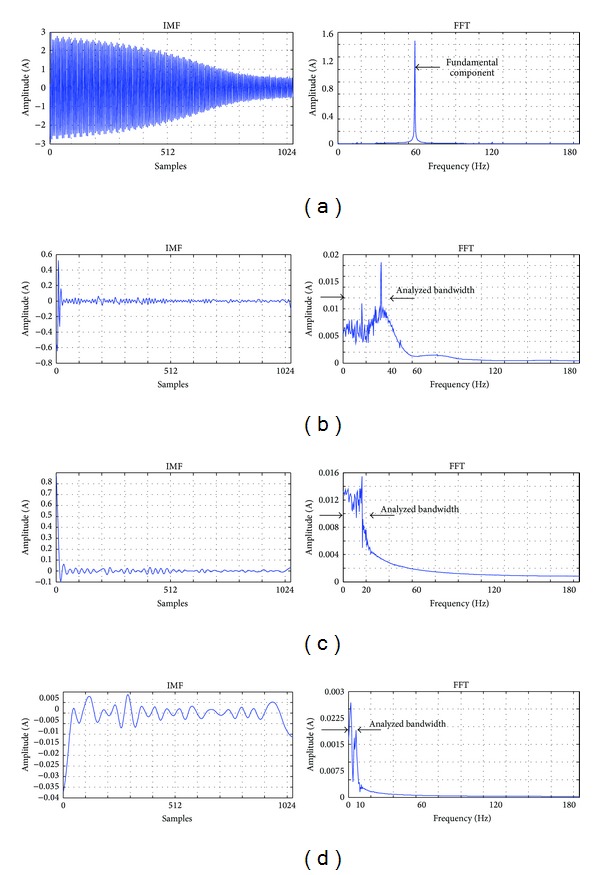
Frequency content for each IMF of a HLT induction motor: (a) first IMF, (b) second IMF, (c) third IMF, and (d) fourth IMF.

The FPGA-based processor is composed by *processing EMD*, *feature extraction ADALINE and classification FFNN* as shown in Figure 5, and the signal processing flow up for a real current sample of a healthy induction motor is depicted in Figure 6. First, the input signal is decomposed by the EMD module in order to obtain the first four IMFs. Notice that for this application, the first IMF has irrelevant information since it contains the 60 Hz fundamental frequency component of the supply system, as is shown in Figure 7(a), and the presence of frequency components related to the motor faults may be undetectable in this bandwidth. Second, third, and fourth IMFs, shown in Figures 7(b), 7(c), and 7(d), respectively, contain within their bandwidths the fault-related frequency components of the motor condition, as summarized in Table 1. The signals *RDD* and *EOD* supervise the incoming data to the EMD unit that computes the IMFs sequentially, and then transfers the result to the ADALINE unit. The frequency-component estimation is done by ADALINE for the second (*H_IMF*_*2*_), third (*H_IMF*_*3*_), and fourth (*H_IMF*_*4*_) IMF, one by one (*IMF_Sel*), and the twelve frequency components (four from each IMF) are stored in the *feature register* unit. Then, the FFNN unit performs the diagnosis by using these frequency components indicating through five outputs the motor condition as: HLT, 1BB, 2BB, UNB, and BD.

### 3.3. EMD Digital Structure

**Figure 8:**
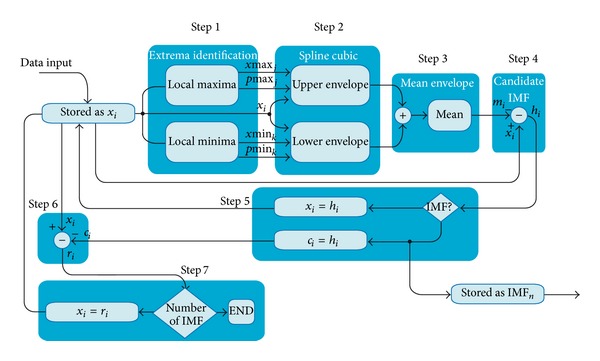
Block diagram of the EMD approach proposed.

Figure 8 shows the required steps for implementing the EMD method. First, the *data input* signal is stored in an internal RAM block as *x*_*i*_, where *i* is 1,2, 3,…, 1024; then, they are sent to *Extrema Identification* module to obtain the extrema of the signal (*x*_max⁡_*j*_, *x*_min⁡_*k*_) and their respective positions (*p*max⁡_*j*_ and *p*min⁡_*k*_). The *spline cubic* module receives *x*_max⁡_*j*_ and *x*_min⁡_*k*_ to calculate the upper and lower envelopes (*S*_*u*_ and *S*_*l*_). The *mean envelope *module calculates the mean of the envelopes (*m*_*i*_). Then, in the* candidate IMF *module the difference between the signal *x*_*i*_ and the signal *m*_*i*_ is calculated in order to obtain the signal *h*_*i*_, which represents a potential IMF. The standard deviation criterion is used to determine if *h*_*i*_ is a true IMF, and if not the signal *h*_*i*_ becomes the input signal and the Steps 1–4 are repeated. When *h*_*i*_ is a true IMF, the signal *h*_*i*_ is stored and becomes the signal *c*_*i*_, then the difference of signals *x*_*i*_ and *c*_*i*_ is defined as *r*_*i*_. Finally, the number of the IMFs calculated is revised to decide if the process ends; if the process goes on, the signal *r*_*i*_ becomes the new input signal and the process is repeated.

#### 3.3.1. Extrema Identification Module

**Figure 9:**
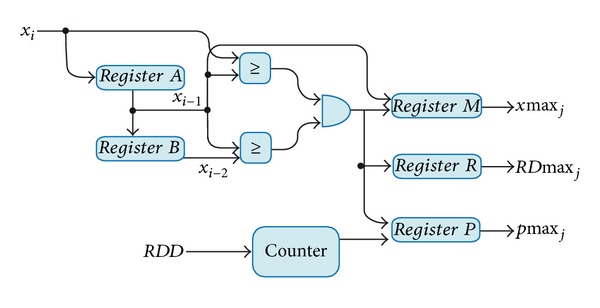
Block diagram of the extrema identification module.

According to Figure 9, first, input *x*_*i*_ is sent to a 2-level pipeline register to store *x*_*i*−1_ in the *register A* and *x*_*i*−2_ in the *register B*. The signal *x*_*i*−1_ is compared with *x*_*i*_ and *x*_*i*−2_ by two comparators, whose results pass through an AND gate. If *x*_*i*+1_ is the greater datum, then it is defined as a maximum, which is placed in *register M *as *x*max⁡_*j*_. The output of the *AND* gate enables *register M*, *register R*, and *register P.* The signal *RD*max⁡ indicates when a new maximum appears. Simultaneously, a *counter *increases by one in every new datum, whereas *register P* stores the position of the maximum (*p*max⁡_*j*_). The same process is used to calculate the minimum, but in this case the comparators are set to detect when *x*_*i*−1_ is smaller than *x*_*i*_ and *x*_*i*−2_.

#### 3.3.2. Spline-Cubic Interpolation Module

**Figure 10:**
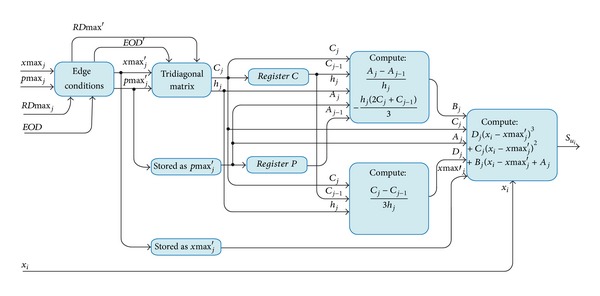
Block diagram of the spline-cubic interpolation module.

The spline-cubic algorithm requires a determined number of data to obtain a smooth interpolation; in this design the size of the selected data set is 1024. After the data set is captured, it is possible to calculate the envelope.  Figure 10 describes the module of spline cubic for calculating of the upper envelope. A similar procedure is used to calculate the lower envelope.

According to Figure 10, the inputs *x*max⁡_*j*_ and *p*max⁡_*j*_ pass through the *edge conditions* module, which adheres the edge conditions in the beginning and ending of the data set of maxima. The signal *RD*max⁡ indicates the arrival of a new maximum and the signal* EOD* the end of the data set. These conditions are used to calculate values on the edges of the signal in the interpolation process. The new data sets (*x*max⁡_*i*_′ and *p*max⁡_*i*_′) with the edge condition are stored and sent to *tridiagonal matrix* module with their respective control signals (*R*max⁡′ and EOD′).

The algorithm of the spline-cubic interpolation according to (15) is

(15) $$\begin{array}{c} S\left(x\right)=\left\{S_{j}\left(x\right),\;x\in \left[x_{j},x_{j+1}\right],\;j\in \left\{0,\ldots ,n-1\right\}\right\}, \end{array}$$

where

(16) Sj(x)=Dj(x−xmax⁡j)3+Cj(x−xmax⁡j)2+Bj(x−xmax⁡j)+Aj.

To obtain the parameter *C*_*j*_, the *tridiagonal matrix *module solves a tridiagonal system for *n* number of maxima with spline natural condition, which is defined by (17) and (18):

(17) [1000⋯0h02(h0+h1)h100h12(h0+h1)h2⋮⋮⋱⋱⋱hn−22(hn−2+hn−1)hn−10⋯001]  ×[C0C1C2Cn−1Cn]=[03(λ1−λ0)3(λ2−λ1)3(λn−1−λn−2)0],

where

(18) $$\begin{array}{c} h_{j}=p\max_{j}-p\max_{j-1},\\ \lambda_{j}=\frac{x\max_{j}-x\max_{j-1}}{h_{j}}. \end{array}$$

After obtaining the value of the coefficient *C*, it is possible to find the values of the coefficients *A*, *B*, and *D* following (19) and making it possible to calculate the envelope

(19) $$\begin{array}{c} A_{j}=p\max_{j}^{\prime },\\ B_{j}=\frac{A_{j}-A_{j-1}}{h_{j}}-\frac{h_{j}\left(2C_{j}+C_{j-1}\right)}{3},\\ D_{j}=\frac{C_{j}-C_{j-1}}{3h_{j}}. \end{array}$$

### 3.4. ADALINE Digital Structure

**Figure 11:**
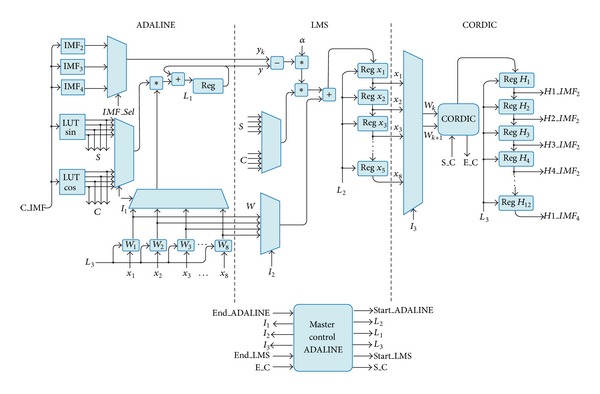
Block diagram of the ADALINE module.

This module performs the frequency estimation for the second (*IMF*_*2*_), third (*IMF*_*3*_), and fourth IMF (*IMF*_*4*_) according to the signal *C_IMF* as shown in Figure 11. The *master Control ADALINE *module provides the overall synchronization to compute (8), (10), (12), and (13) for ADALINE (*Start_ADALINE*/*End_ADALINE*), LMS (*Start_ADALINE*/*End_ADALINE*), and CORDIC (*S_C*/*E_C*). First, the ADALINE section performs (8) as follows: the sine and cosine values for only one period of the twelve frequency components are stored in the lookup tables (LUT) *LUT sin* and *LUT cos*, respectively; afterwards, they are multiplied and added by the weights stored in the registers *W*_*i*_ for *i* = 0,…, 8 since there are four frequency components and each one of them requires the coefficients *a* and *b* according to (9). Second, the LMS section computes (13) in order to minimize the error between the ADALINE output *y* and the desired output *y*_*k*_. For this, the error in (12) is first computed and multiplied by *α*; in this methodology, *α* = 0.01 is used. Posteriorly, the modified weights are stored in the registers *X*_*i*_ for *i* = 0,…, 8 through the multiplication and summation of *S*, *C*, and *W*_*i*_, which are the sine, cosine, and weights values, respectively. Finally, the coordinate rotation digital computer (*CORDIC) *section estimates the amplitude and phase according to (10) [42]. It is used in vectoring mode, where the weights are taken in pairs, *W*_*k*_ and *W*_*k*+1_. In order to obtain the twelve frequency components (*H1_IMF*_*2*_, *H2_IMF*_*2*_, *H3_IMF*_*2*_, *H4_IMF*_*2*_,…,* H4_IMF*_*4*_), this overall process is repeated for the three IMFs. The signals *I*_*i*_ and *L*_*i*_ for *i* = 1,2, and 3 control the multiplexers and the registers load, respectively.

### 3.5. FFNN

**Figure 12:**
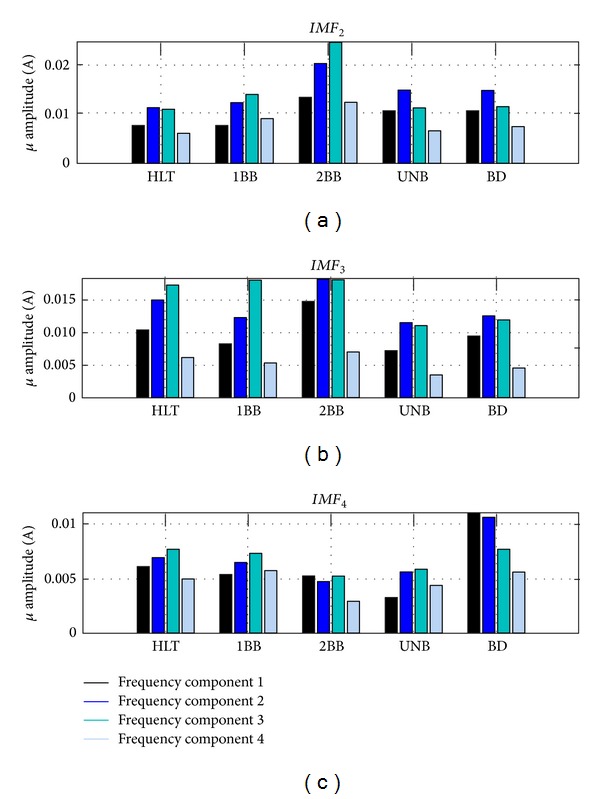
Frequency components estimation through ADALINE for different faults.

**Figure 13:**
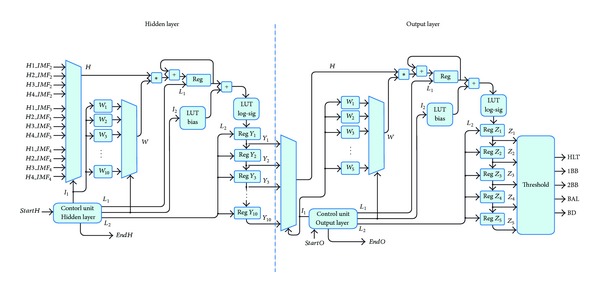
Block diagram of the FFNN module: (a) hidden layer, and (b) output layer.

This module is first developed and trained in Matlab for being subsequently implemented in the FPGA. Therefore, the FFNN module is firstly trained through the Levenberg-Marquardt algorithm for identifying a HLT condition in the induction motor or the presence of multiple single faults. For this, twenty real sampled signals are carried out under each motor condition. The training and validation sets for each condition are obtained synthetically by randomly producing 100 values, 70 for training and 30 for validation, of each frequency component within the range (*μ* + *σ*, *μ* − *σ*), where *μ* is the mean and *σ* is the standard deviation of the frequency components magnitude from the twenty real sampled signals, as shown in Figure 12. The testing set is composed by real signals only. The FFNN final architecture has 12 inputs (four frequency components for each IMF), 10 neurons in the hidden layer, and 5 outputs (one per each condition), that function as flags to indicate the induction motor condition. The number of 10 neurons in the hidden layer is selected by trial and error in order to obtain the minimum overall classification error. After the training, validation, and testing, the final weights and biases of each layer neuron are used for FPGA implementation according to the digital structure shown in Figure 13, which computes (14) for each neuron. There, the *control unit hidden *and* output layers* provide the overall synchronization signals that regulate the information exchange among the control units for the hidden and output layers through *StartH*/*EndH* and *StartO*/*EndO*, respectively; besides, the signals *I*_*i*_ and *L*_*i*_ for *i* = 1 and 2 control the multiplexers and the registers load. The hidden layer shown in Figure 13(a) has 10 neurons and receives the four frequency components for each IMF. They are weighted by the corresponding values *W*_1_, *W*_2_,…, *W*_10_. Each *W*_*k*_ register contains 12 different weighted values, one for each input. The weighted values for each frequency component are summed up and added sequentially to a bias value stored in a LUT (*LUT bias*). The result of this operation is used for triggering on the respective output *Y*_*i*_ through a log-sigmoid (LS) transfer function, which is implemented as a LUT (*LUT log-sig*). The same process is repeated in the output layer shown in Figure 13(b), which uses the outputs *Y*_1_, *Y*_2_,…, *Y*_10_ from the hidden layer as inputs to its five neurons obtaining the outputs *Z*_1_, *Z*_2_,…, *Z*_5_ that define HLT, 1BB, 2BB, UNB, and BD condition through a threshold comparison of 0.5. Thus, the display module shows the induction motor condition according to the activated output neuron.

## 4. Experimentation and Results

This section presents the experimental setup used to test the proposed FPGA-based methodology under real operating conditions as well as the obtained results.

### 4.1. Experimental Setup

**Figure 14:**
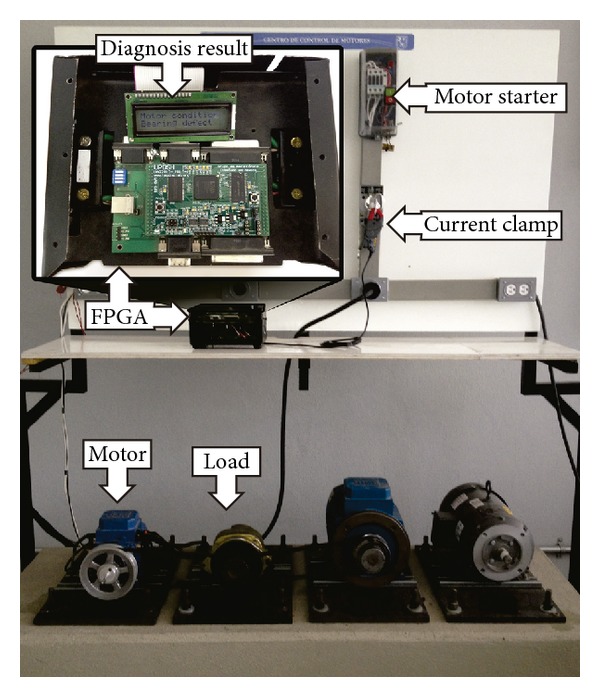
Experimental setup.

Figure 14 shows the experimental setup where 1-hp three-phase induction motor (model WEG00136APE48T) is used to test the proposed FPGA-based methodology. The tested motors have two poles, 28 bars, and receive a power supply of 220 Vac at 60 Hz. The mechanical load is an ordinary alternator. One phase of the stator current is acquired with an i200 Fluke current clamp. The DAS has a sampling frequency of 375 Hz, taking 1024 samples during the startup transient. The information is transferred to the FPGA processor to perform the motor diagnosis through EMD, ADALINE, and FFNN. Finally, the diagnosis result is shown in the display.

### 4.2. Treated Faults

**Figure 15:**
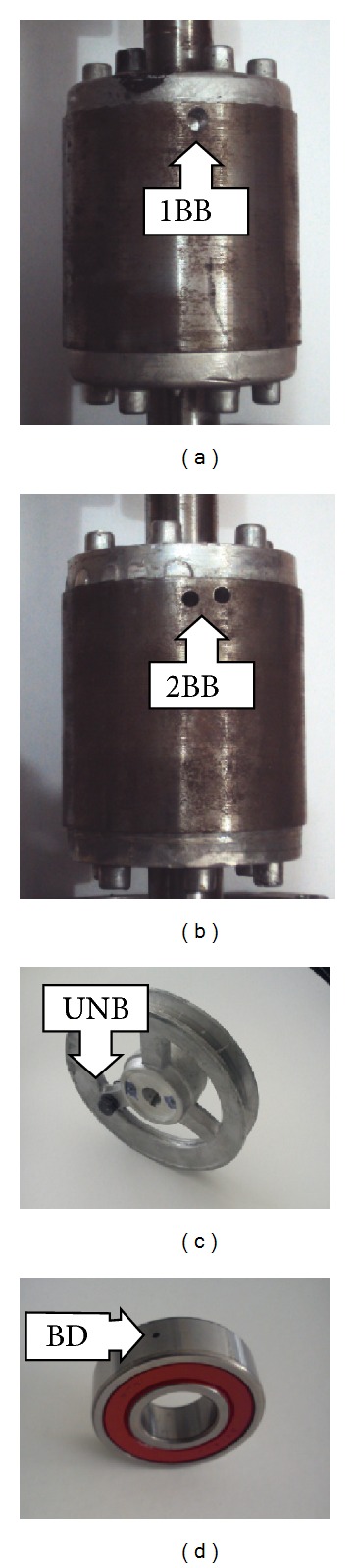
Treated faults.

The broken rotor bar condition is artificially produced by drilling an 8 mm diameter hole without harming the rotor shaft.  Figure 15(a) shows a rotor with one broken bar (1BB) and Figure 15(b) depicts a rotor having two broken bars (2BB). The UNB condition appears when the mechanical load is not uniformly distributed in the induction motor with the center of mass displaced from the motor shaft.  Figure 15(c) shows a pulley with an added eccentric mass for generating unbalance in the motor shaft. The bearing defect (BD) is produced by drilling a hole of 1.2 mm diameter on its outer race, as shown in Figure 15(d).

### 4.3. Results

The FPGA-based system uses 18-bit fixed-point arithmetic, which generates rounding and truncation errors. In order to evaluate the overall performance of the developed FPGA implementation, the fixed-point results are compared against floating-point Matlab simulations for the same acquired data sets.  Table 2 presents the mean (*μ*), the standard deviation (*σ*), and the peak (*P*) of relative errors when comparing fixed-point (FPGA) and floating-point (Matlab) results for the 20 trials of experimental data under each motor condition, where the worst values are indicated in bold, being 1BB condition.

**Figure 16:**
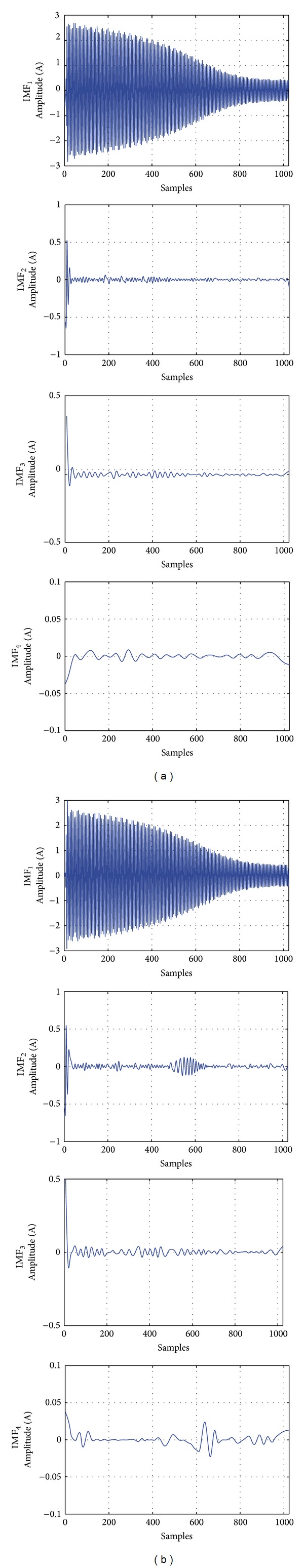
Resulting IMFs of the EMD hardware implementation: (a) healthy motor and (b) faulty motor with two broken rotor bars.

Table 3 shows the classification results as well as the effectiveness percentage of the proposed methodology. The testing set composed by 100 real trials, 20 for each induction motor condition, is classified by the proposal as follows: of the 20 actual HLT conditions, the system classifies 20 HLT conditions; therefore, it has an effectiveness of 100%. On the other hand, of the 20 1BB conditions, the system classifies one as HLT, 18 as 1BB, and one as 2BB; therefore, it has an effectiveness of 90%. All correct classifications are located in the diagonal of Table 3 (highlighted in bold). Particularly, Figure 16 shows the results of the EMD hardware implementation for the HLT and 2BB conditions.

Table 4 summarizes the hardware implementation resources for the proposed methodology. The number of clock cycles taken for the main structures to perform their computation is also presented, where it is important to notice that the number of cycles shown for the EMD is the average of the tests, since the duration of the EMD depends on the signal complexity. The used platform is a proprietary board, based on the Spartan 3E XCS1600 FPGA running at 48 MHz.

Time-computation performance of the EMD implementation can be estimated by the number of clock cycles required to compute a full input data set with *N* samples. In this design for calculating an envelope, the spline-cubic algorithm needs that the data set is fully acquired and then calculates the *N* points of the envelope. This means that it takes 2*NT*_*m*_ clock cycles to calculate a candidate IMF, where *T*_*m*_ is the number of clock cycles required to calculate each sample. *T*_*m*_ is determined by two consecutive division operations that consume 56 clock cycles, which for this implementation of the EMD, the digital structure can reach a peak of 857 kHz of sampling frequency for a 48 MHz master clock. This design uses a data set of 1024 points that would take 114,688 clock cycles to calculate a candidate IMF, or 2.38 ms at 48 MHz. Huang et al. [43] mention that typically there 3 to 4 interactions are required to have an IMF, so the worst case for calculating four IMFs will require 16 interactions that take 1,835,008 clock cycles, or 38.22 ms at 48 MHz.

### 4.4. Analysis and Discussion

The results show that the hardware implementation of the proposed methodology has 100% effectiveness in detecting the HLT, BD, UNB, and 2BB conditions. For the 1BB condition, the percentage of a correct identification is 90% where two results are mistaken with HLT and 2BB conditions.

The low values for the standard deviation and the peak relative errors of the results presented in Table 2 show the feasibility of the proposed FPGA implementation, considering a fixed-point arithmetic approach in the digital structure.

The resource usage presented in Table 4 shows the viability of implementing the EMD, ADALINE, and FFNN structures as well as their fusion in a low-cost SoC solution for induction motor diagnosis. Besides, the FPGA-based proposed methodology takes 1,069,141 clock cycles, equivalent to 22.27 ms at 48 MHz, for estimating the induction motor condition, which outperforms, by one order of magnitude, the Matlab implementation that takes 815.53 ms on a 2.2 GHz Intel Core i7 processor. Another important characteristic is that the proposed EMD digital structure can support sampling rates of up to 857 kHz, implying the potential of further hardware implementations for online applications like biosignal processing, power quality estimation, and acoustic and seismic analysis, whose signals work below this sampling rate.

Table 5 shows a comparison of the main characteristics between the reported works in the literature and the one proposed here. Regarding the hardware implementation, most works are PC-based, which can compromise the online operation, and only this work and references [26, 27] present a FPGA solution; yet, the proposal is able to process stationary and transient signals unlike the aforementioned works; in addition it does not need a previous design since EMD is an adaptive and unsupervised technique. Moreover, it has the option to send the data for PC after processing as done in other reported works and systems. On the other hand, the number of faults that can be detected through the proposed methodology is greater than the other ones that also use the EMD technique.

## 5. Conclusions

This paper presents a new online novel digital structure to implement the EMD method in FPGA using the full spline-cubic function and not just a linear approximation, taking into account that the spline-cubic function does not present the problem of high-frequency component rides, making it more suitable for time-frequency processing. This novel structure for the hardware computation of EMD method is combined with an ADALINE-based frequency estimator and an ANN classifier in the same FPGA to provide an intelligent methodology for online multiple fault diagnosis in induction motors. First, the startup transient current signal, which is non-stationary, is processed by the EMD in order to obtain the IMFs; then, the ADALINE estimates the magnitude of only four frequency components distributed symmetrically in a bandwidth estimated for the second, third, and fourth IMF. Finally, these frequency components are used as input features of an ANN classifier for the automatic diagnosis of motor faults such as: one (1BB) and two broken rotor bars (2BB), bearing defects (BD), and unbalance (UNB). Besides, the overall methodology implementation into an FPGA allows an online and real-time operation, thanks to its parallelism and high-performance capabilities as a system-on-a-chip (SoC) solution, unlike other works where results have to be interpreted offline by the user from the current or vibration signals.

On the other hand, the high precision and minimum resource usage of the proposed and developed digital structures make them attractive for many other applications, highlighting that the novel EMD digital structure uses the full spline-cubic function and not only a linear approximation, avoiding the presence of problems like high-frequency component rides, as cited in literature, making it more suitable for time-frequency processing of non-stationary signals in industrial applications.

## Figures and Tables

**Table 1:** Selected frequency components for each IMF.

IMF	Analyzed bandwidth (Hz)	Selected frequency components (Hz)
2	0–40	10, 20, 30, 40
3	0–20	5, 10, 15, 20
4	0–10	2.5, 5, 7.5, 10

**Table 2:** FPGA-based implementation performance (the worst values are highlighted in bold).

Induction motor condition	Relative error (%)		
	Mean (*µ*)	Standard deviation (*σ*)	Peak error (*P*)
HLT	0.4291	0.0743	0.5638
1BB	**0.6146**	**0.0960**	**0.7902**
2BB	0.1780	0.0564	0.3083
BD	0.4025	0.0803	0.5706
UNB	0.2091	0.0768	0.3518

**Table 3:** Effectiveness percentage of the proposed methodology (confusion matrix).

	HLT	1BB	2BB	BRN	UNB	Effectiveness (%)
HLT	**20**	0	0	0	0	100
1BB	1	**18**	1	0	0	90
2BB	0	0	**20**	0	0	100
BD	0	0	0	**20**	0	100
UNB	0	0	0	0	**20**	100

**Table 4:** Resource usage of the FPGA.

Resource utilization	EMD	ADALINE	FFNN	Total used	Available	Percentage
Programmable logic	3,731	3,198	1,582	8,511	29,504	28.85
LUTS	11,211	7,437	4,795	23,443	29,504	79.45
Multipliers	30	4	2	36	36	100

Clock cycles	984,564	84,340	237	Total	1,069,141	

**Table 5:** Main characteristics of previous works and of the proposed work.

Work	Faults	Methodology based on	*A priori* design required		Signals		Hardware	
			Yes	No	S	T	PC	FPGA
[12–16]	Bearings	EMD		X	X	X	X	

[17–22]	Broken bar	EMD		X	X	X	X	

[22–25]	Rotor eccentricities	EMD		X	X	X	X	

[26]	Broken bar Unbalance Misalignment	FFT	X			X	X	X

[27]	Broken bars Unbalance Looseness	FFT	X			X	X	X

This work	Broken bars Bearings Unbalance	EMD		X	X	X	X	X

S: stationary; T: transient.
